# Dynamic Transcriptomic Analysis of Breast Muscle Development From the Embryonic to Post-hatching Periods in Chickens

**DOI:** 10.3389/fgene.2019.01308

**Published:** 2020-01-10

**Authors:** Jie Liu, Qiuxia Lei, Fuwei Li, Yan Zhou, Jinbo Gao, Wei Liu, Haixia Han, Dingguo Cao

**Affiliations:** ^1^Poultry Institute, Shandong Academy of Agricultural Sciences, Jinan, China; ^2^Poultry Breeding Engineering Technology Center of Shandong Province, Jinan, China; ^3^Shandong Provincial Key Laboratory of Poultry Diseases Diagnosis and Immunology, Jinan, China

**Keywords:** breast muscle, chickens, development, differential gene expression, RNA sequencing

## Abstract

Skeletal muscle development and growth are closely associated with efficiency of poultry meat production and its quality. We performed whole transcriptome profiling based on RNA sequencing of breast muscle tissue obtained from Shouguang chickens at embryonic days (E) 12 and 17 to post-hatching days (D) 1, 14, 56, and 98. A total of 9,447 differentially expressed genes (DEGs) were filtered (*Q* < 0.01, fold change > 2). Time series expression profile clustering analysis identified five significantly different expression profiles that were divided into three clusters. DEGs from cluster I with downregulated pattern were significantly enriched in cell proliferation processes such as cell cycle, mitotic nuclear division, and DNA replication. DEGs from cluster II with upregulated pattern were significantly enriched in metabolic processes such as glycolysis/gluconeogenesis, insulin signaling pathway, calcium signaling pathway, and biosynthesis of amino acids. DEGs from cluster III, with a pattern that increased from E17 to D1 and then decreased from D1 to D14, mainly contributed to lipid metabolism. Therefore, this study may help us explain the mechanisms underlying the phenotype that myofiber hyperplasia occurs predominantly during embryogenesis and hypertrophy occurs mainly after birth at the transcriptional level. Moreover, lipid metabolism may contribute to the early muscle development and growth. These findings add to our knowledge of muscle development in chickens.

## Introduction

In chicken production, skeletal muscle development is closely associated with the amount of meat production and its quality, ultimately affecting the economic benefits. Therefore, elucidating the molecular mechanisms underlying chicken skeletal muscle development is of vital interest. The muscle mass is determined by cell numbers and unit cell size. Hyperplasia refers to the increases in cell number or muscle fiber number that occur mainly in the embryonic period, as the number of muscle fibers is fixed by the day of hatching. However, hypertrophy refers to the increase in cell size that occurs mainly after birth (Ylihärsilä et al., 2007; Liu et al., 2017b; Ouyang et al., 2017). Therefore, there may be distinct molecular processes that occur in chicken muscle development between the embryonic and post-hatching periods.

Over the past few years, there has been much progress in exploring the molecular mechanisms underlying muscle growth and development in chickens, but most of the studies focused on the embryonic or post-hatching period. Davis et al. (2015) characterized the transcriptome of Ross 708 chicken breast muscle at specified time points from 6 to 21 days after hatching. Li et al. (2019) explored the messenger RNA (mRNA) and microRNA (miRNA) profiles of Gushi chicken muscle tissues in the late postnatal stage (6, 14, 22, and 30 weeks). Our previous study examined the protein expression profiles in the breast muscle of Beijing-You chickens at ages 1, 56, 98, and 140 days using isobaric tags for relative and absolute quantification (Liu et al., 2016). Li et al. (2017) and Ouyang et al. (2017) explored the transcriptome and protein expression profiles in leg muscle tissues of Xinghua chicken at embryonic days (E) 11 and E16 and post-hatching day (D) 1, respectively. However, few studies have paid attention to the whole muscle development from embryonic to post-hatching periods in chickens. Only Liu et al. (2017b) investigated the proteomes of breast muscle in Cobb and Beijing-You chickens at E12, E17, D1, and D14.

Shouguang chickens that have been breed in China for 2,000 years are a dual-purpose breed with large bodies (Gao et al., 2008), which may be excellent material for studying muscle development. Therefore, we chose the critical breast muscle developmental stages in the embryonic to post-hatching periods (E12 and E17 and D1, D14, D56, and D98) of Shouguang chickens for quantitative analysis of the gene expression profile of breast muscle, which may help us explore the development-related genes expression signatures in breast muscle and its distinction between embryonic and post-hatching periods.

## Methods

### Animals

Shouguang chicken eggs were obtained from the experimental farm of the Poultry Institute (PS), Shandong Academy of Agricultural Sciences (SAAS, Jinan, China). All eggs were incubated with the normal procedure and chicks were reared in cages using standard conditions of temperature, humidity, and ventilation at the farm of the PS, SAAS. The same diet was fed to all chickens and a three-phase feeding system was used: starter ration (days 1–28) with 21.0% crude protein and 12.12 MJ/kg; second phase (days 28–56) with 19.0% crude protein and 12.54 MJ/kg; and final phase (after day 56) with 16.0% crude protein and 12.96 MJ/kg. Feed and water were provided *ad libitum* during the experiment. Breast muscles were used at E12, E17, D1, D14, D56, and D98. All fresh breast muscle tissue samples were collected, frozen in liquid nitrogen, and stored at −80°C until RNA extraction. The sex of chicken embryos was identified by polymerase chain reaction (PCR) of the *CHD1* gene (Fridolfsson and Ellegren, 1999). Chickens with two bands of 600 and 450 bp were born as female, and those with one band of 600 bp were born as male.

### RNA Extraction, cRNA Library Construction, and Sequencing

Total RNA was extracted using TRIzol reagent (Invitrogen, Carlsbad, CA, USA). Three female chickens at each stage (except E17) were used for further experiments. Total RNA quantity and purity were analyzed using a Bioanalyer 2100 (Agilent, Santa Clara, CA, USA) with RNA integrity number >7.0. Approximately 10 μg total RNA was used to deplete rRNA using the Epicentre Ribo-Zero Gold Kit (Illumina, San Diego, CA, USA). Following purification, the poly(A)− or poly(A)+ RNA fraction was fragmented into small pieces using divalent cations under elevated temperature. The cleaved RNA fragments were reverse-transcribed to create the final complementary DNA (cDNA) library in accordance with the protocol for the RNA sequencing (RNA-Seq) sample preparation kit (Illumina). The average insect size for the paired-end libraries was 300 ± 50 bp. We performed paired-end sequencing on an Illumina Hiseq 4000 at LC-Bio, China.

### RNA-Seq Reads Mapping and DEG Analysis

We aligned reads to the genome of Gallus_gallus 5.0 (GCA_000002315.3) using HISAT package (Kim et al., 2015), which initially removed reads based on quality information accompanying each read and then mapped the reads to the reference genome. The mapped reads of each sample were assembled using StringTie (Pertea et al., 2015). All transcriptomes from samples were merged to reconstruct a comprehensive transcriptome using perl scripts. After the final transcriptome was generated, StringTie and edgeR (Robinson et al., 2010) were used to estimate the expression levels of all transcripts. StringTie was used to perform expression level for mRNAs by calculating fragments per kilobase of transcript per million fragments mapped (FPKM). Differentially expressed genes (DEGs) were selected with log2 (fold change) > 1 or log2 (fold change) less than −1 with statistical significance (*Q* < 0.01) by R package. The raw sequence data reported in this paper have been deposited in the Genome Sequence Archive in BIG Data Center, Beijing Institute of Genomics (BIG), Chinese Academy of Sciences, and is publicly accessible at http://bigd.big.ac.cn/gsa (accession no. CRA001773).

### Time Series Expression Profile Clustering

The non-parametric clustering algorithm of STEM (Short Time-Series Expression Miner, version 1.3.11) (Ernst and Bar-Joseph, 2006) was used to cluster and visualize the expression patterns of DEGs. Expression profiles of DEGs were clustered based on their log2 (FPKM values) and their correlation coefficients. The maximum unit change in model profiles between time points was adjusted to 2 and the maximum number of model profiles to 50. The statistical significance of the number of DEGs to each profile versus the expected number was computed using the algorithm proposed by Ernst and Bar-Joseph (2006).

### Functional Annotation

Functional analysis of DEGs was performed using the DAVID (Database for Annotation, Visualization and Integrated Discovery) tool (http://david.abcc.ncifcrf.gov/) (Dennis et al., 2003). The Kyoto Encyclopedia of Genes and Genomes (KEGG) pathway analysis was performed using KOBAS version 3.0 (Xie et al., 2011). Gene Ontology (GO) terms and KEGG pathways with *P* < 0.05 were considered significantly enriched groups of genes possibly contributing to muscle development.

### qRT-PCR Confirmation

To confirm our differential expression results, we conducted quantitative reverse transcription PCR (qRT-PCR) for six selected genes (*MYOG*, *MYH11*, *TNNI2*, *TNNT3*, *TNNC2*, and *TPM2*). The total RNA was used for first-strand cDNA synthesis using a commercial kit (TaKaRa, Dalian, China). cDNA was subsequently used for qRT-PCR analyses with an ABI 7500 Detection System (Applied Biosystems, Foster City, CA, USA) and primers designed using Primer Premier version 5.0 (PREMIER Biosoft, Palo Alto, CA, USA), as listed in **Table S1**. mRNA abundance of candidate genes was determined using the KAPA SYBR^®^ FAST qPCR Master Mix (2×) Universal Cocktail (KAPA Biosystems, Boston, MA, USA). qRT-PCR was performed following the instructions of ABI 7500 with default parameters. The 2^−ΔΔCt^ method (Livak and Schmittgen, 2001) was used to calculate the relative mRNA abundance. The beta actin gene (*ACTB*) was used as the housekeeping gene. Three independent replications were used for each assay and data were presented as means ± SD.

## Results

### Overall Assessment for Sequencing Data Mapping Statistics

To identify mRNA expressed in breast muscle tissue development of chickens, we constructed 17 cDNA libraries (E12_1, E12_2, E12_3, E17_1, E17_2, D1_1, D1_2, D1_3, D14_1, D14_2, D14_3, D56_1, D56_2, D56_3, D98_1, D98_2, and D98_3) from breast muscle samples at six developmental stages. As shown in **Table 1**, 68,431,306–103,358,850 raw reads were generated in the 17 libraries, and 65,384,366–100,882,250 clean reads were obtained after discarding adaptor sequences and low-quality reads. We mapped clean reads to chicken reference genome Gallus_gallus 5.0 and found that 84.41–90.10% of the clean reads in the libraries were mapped to the chicken reference genome (**Table 1**).

**Table 1:** Overview of raw data output and quality assessment.

Sample	Raw reads	Clean reads	Mapped reads	Mapped rate (%)
E12_1	75,233,696	72,137,830	63,804,708	88.45
E12_2	71,868,594	68,692,864	60,781,832	88.48
E12_3	73,728,758	70,509,198	61,614,305	87.38
E17_1	76,625,816	73,423,116	65,037,045	88.58
E17_3	68,431,306	65,384,366	56,147,755	85.87
D1_1	75,819,908	71,918,030	61,870,757	86.03
D1_2	87,249,520	80,288,708	71,793,909	89.42
D1_3	75,927,124	71,486,238	60,974,309	85.30
D14_1	88,752,924	86,540,700	77,973,761	90.10
D14_2	81,110,076	79,280,896	69,851,415	88.11
D14_3	88,685,236	86,822,844	74,453,649	85.75
D56_1	86,677,672	84,671,272	74,896,179	88.46
D56_2	88,218,208	86,364,222	75,789,517	87.76
D56_3	96,7881,00	94,729,644	82,666,195	87.27
D98_1	96,819,270	94,753,276	83,684,517	88.32
D98_2	103,358,850	100,882,250	86,849,158	86.09
D98_3	89,320,116	87,415,674	73,785,643	84.41

### Differential Expression Analysis of Genes

**Figure 1:**
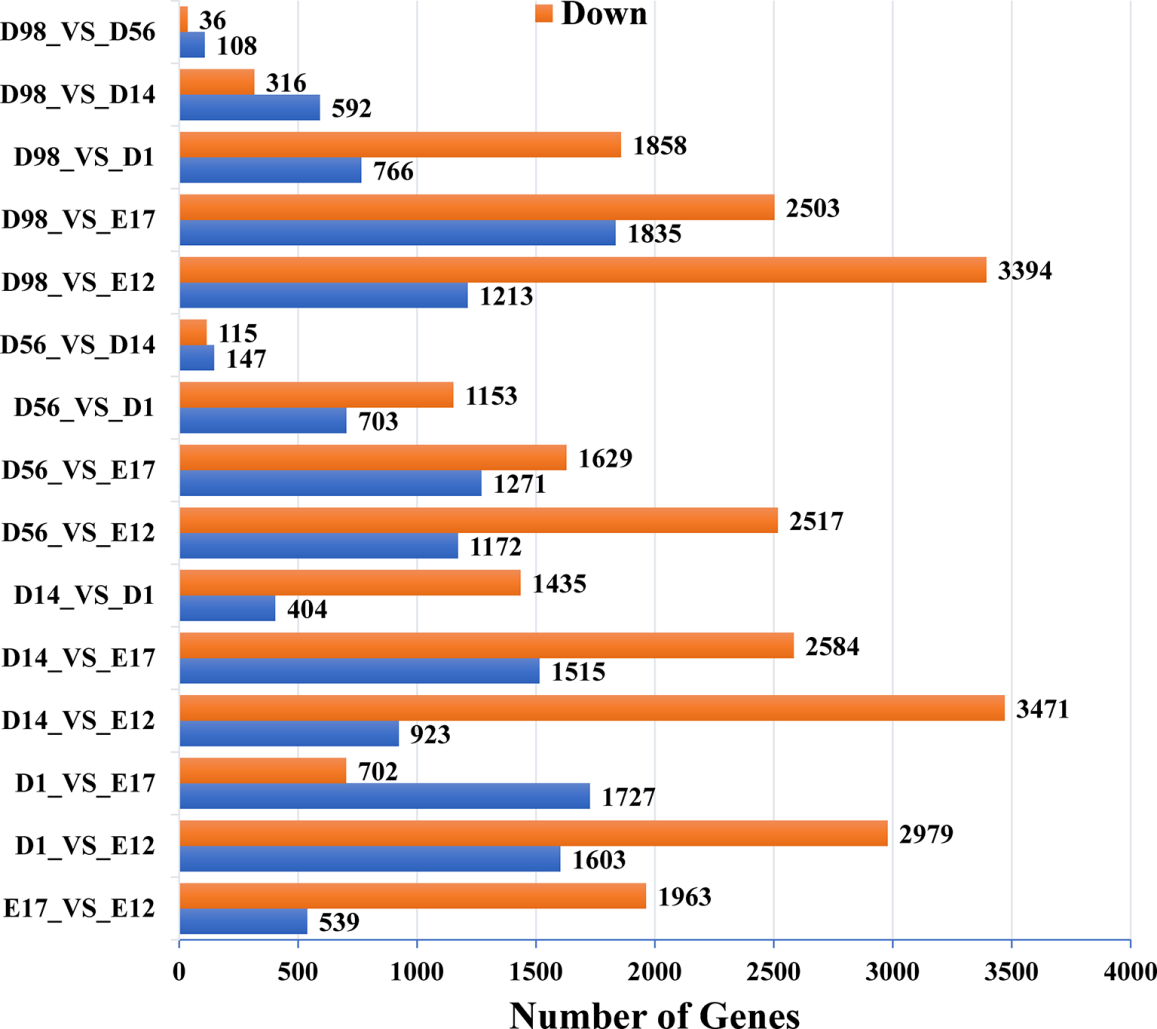
Numbers of upregulated and downregulated genes in chicken breast muscle through pairwise comparisons.

In pairwise comparisons between the libraries of breast muscle at the six developmental stages, a total of 9,447 genes were differentially expressed (*Q* < 0.01, fold change > 2) (**Figure 1** and **Table S2**). There were 2,502, 4,582, 4,394, 3,689, and 4,607 DEGs in E17, D1, D14, D56, and D98 compared to E12. Comparing successive ages within each region, 2,502, 2,429, 1,839, 262, and 144 DEGs were found in E17 versus E12, D1 versus E17, D14 versus D1, D56 versus D14, and D98 versus D56, respectively. The numbers of DEGs were greatest in E17 versus E12 and lowest in D98 versus D56, which indicated that regional differences in gene expression were greatest during the earlier stages of embryo development.

### STEM Analysis of DEG Profiles

**Figure 2:**
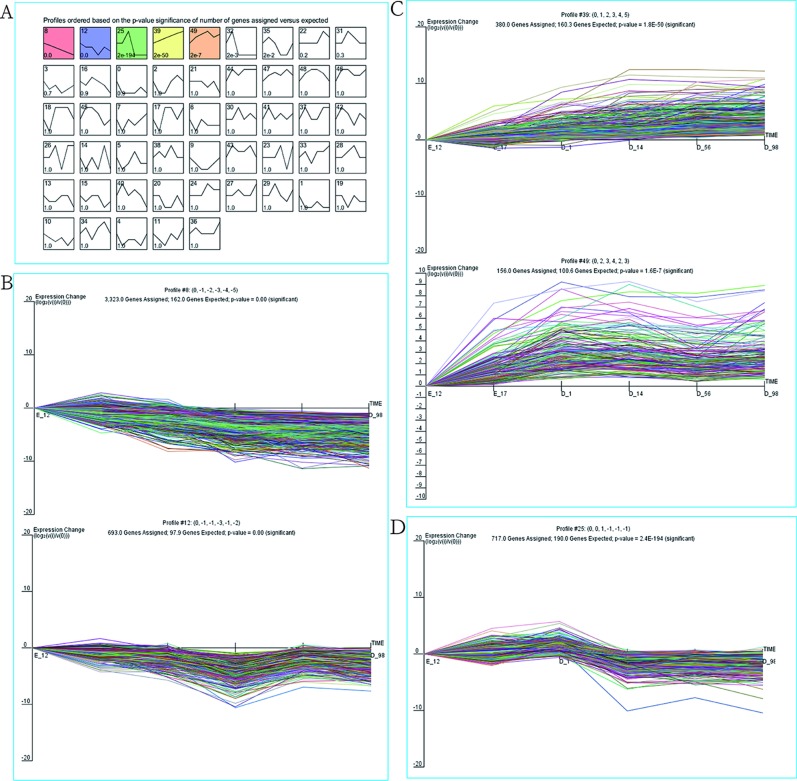
STEM analysis of DEG profiles. **(A)** Each *box* corresponds to a type expression profile and only *colored profiles* are significantly different. The *upper-left* and *upper-right numbers in each box* indicate the order of profiles and *P* values, respectively. **(B)** Profile 8 (up) and profile 12 (down) with downregulated patterns. **(C)** Profile 39 (up) and profile 49 (down) with upregulated patterns. **(D)** Profile 25.

As our data were collected at different time points, STEM was used to cluster and visualize possible changes in the profiles of 9,447 DEGs at six time points of breast muscle development. Within the 50 model profiles, five expression profiles containing 5,269 genes were statistically significant (*P* < 0.05; **Figure 2A** and **Table S3**). Of these, profiles 8 and 12 with downregulated patterns contained 3,233 and 693 DEGs, respectively (**Figure 2B** and **Table S3**), while profiles 39 and 49 with upregulated patterns contained 380 and 156 DEGs, respectively (**Figure 2C** and **Table S3**). Profile 25 with 717 genes as the third pattern showed an increase from E17 to D1 and reached a peak at D1, then decreased from D1 to D14 and remained stable from D14 to D98 (**Figure 2D** and **Table S3**). Thus, the expression pattern of DEGs can be divided into three clusters: cluster I (profiles 8 and 12, total of 3,926 DEGs) with downregulated pattern; cluster II (profiles 39 and 49, total of 536 DEGs) with upregulated pattern; and cluster III (profile 25, total of 717 DEGs). The results provide new information related to further characterization of novel molecules associated with skeletal muscle development in chickens.

### GO Enrichment Analysis

**Figure 3:**
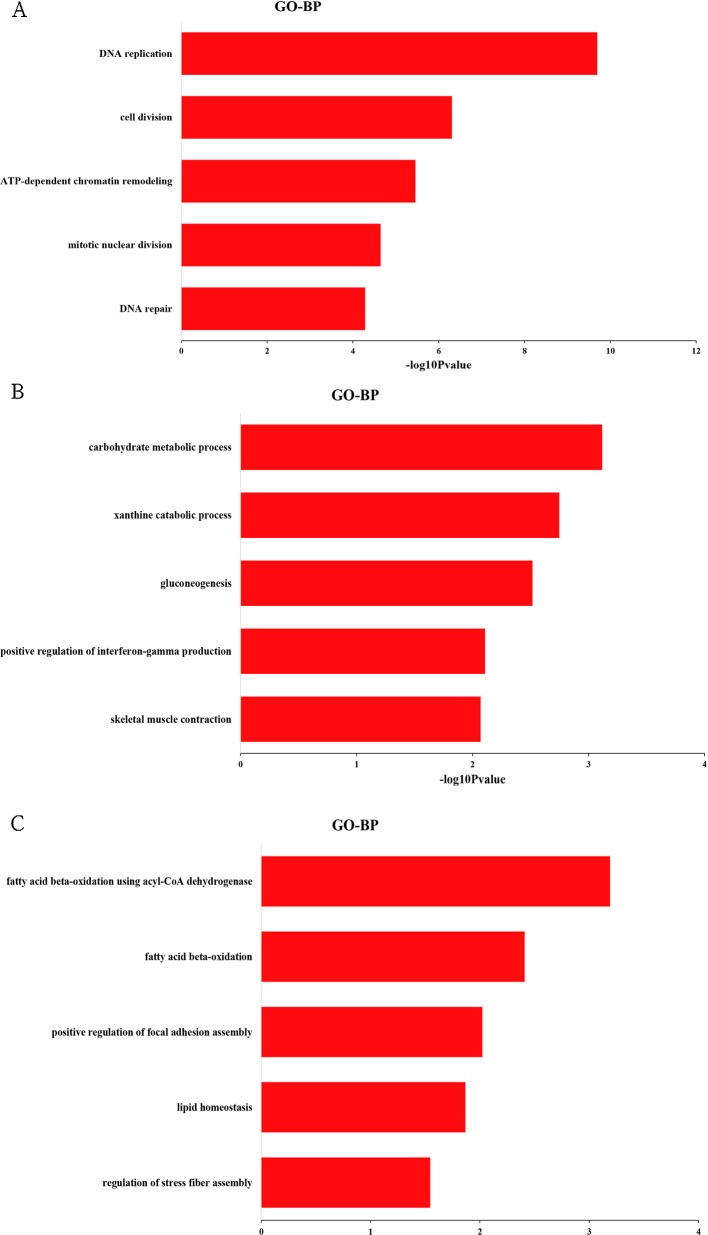
Top 5 significantly enriched biological process terms of genes in cluster I **(A)**, cluster II **(B)**, and cluster III **(C)**.

To explore the biological function of DEGs, GO enrichment analysis was performed based on cluster analysis. The genes in cluster I (profiles 8 and 12) were significantly enriched in 138 GO terms (68 under biological process, 38 under cellular component, and 32 under molecular function) (**Table S4**). Within the biological process category, the most abundant GO terms consisted of DNA replication, cell division, ATP-dependent chromatin remodeling, mitotic nuclear division, and DNA repair (**Figure 3A**). The genes in cluster II (profiles 39 and 49) were significantly enriched in 34 GO terms (16 under biological process, six under cellular component, and 12 under molecular function) (**Table S5**). Within the biological process category, the most abundant GO terms consisted of carbohydrate metabolic process, xanthine catabolic process, gluconeogenesis, and positive regulation of interferon-γ production (**Figure 3B**). Skeletal muscle contraction was also included in this category (**Figure 3B**). We also analyzed the biological function of genes in cluster III (profile 25). Eighteen GO terms (10 under biological process, three under cellular component, and five under molecular function) were significantly enriched (**Table S6**). Within the biological process category, the most abundant GO terms consisted of fatty acid beta-oxidation using acyl-CoA dehydrogenase, fatty acid beta-oxidation, positive regulation of focal adhesion assembly, lipid homeostasis, and regulation of stress fiber assembly (**Figure 3C**).

### KEGG Enrichment Analysis

**Figure 4:**
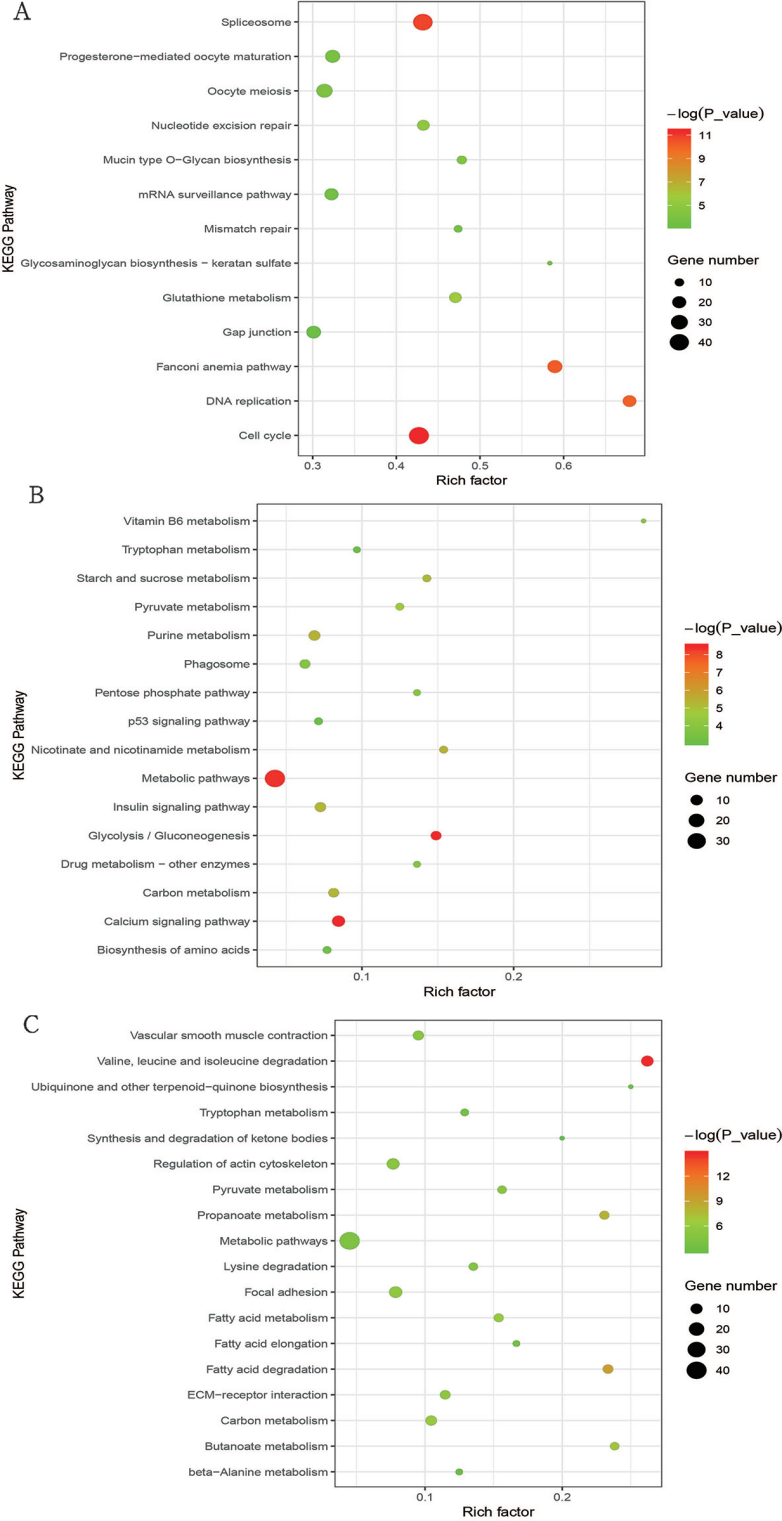
Bubble plot of significantly enriched pathways for cluster I (profiles 8 and 12) with downregulated pattern **(A)**, cluster II (profiles 39 and 49) with upregulated pattern **(B)**, and cluster III (profile 25) **(C)**. *Bubble color* and *size* correspond to the *P* value and gene number enriched in the pathway. The rich factor indicates the ratio of the number of DEGs mapped to a certain pathway to the total number of genes mapped to this pathway.

We used KEGG pathway analysis to explore the signaling pathways of DEGs involved in cluster I (profiles 8 and 12), cluster II (profiles 39 and 49), and cluster III (profile 25). For cluster I, the DEGs were significantly enriched in 13 pathways (**Figure 4A** and **Table S7**), and half of these pathways are involved in cell division, such as cell cycle, DNA replication, nucleotide excision repair, mismatch repair, oocyte meiosis, and spliceosome. As for cluster II, the DEGs were significantly enriched in 16 pathways (**Figure 4B** and **Table S8**), and it was interesting that all of these pathways were directly or indirectly involved in the main metabolic processes of the organism, such as glycolysis/gluconeogenesis, purine metabolism, starch and sucrose metabolism, vitamin B6 metabolism metabolic pathways, pentose phosphate pathway, biosynthesis of amino acids nicotinate, and insulin signaling pathway. In cluster III, the DEGs were significantly enriched in 18 pathways (**Figure 4C** and **Table S9**), and most of these pathways were associated with metabolism. Among these metabolic pathways, the most enriched pathways were those related to lipid metabolism, such as fatty acid degradation, propanoate metabolism, butanoate metabolism, fatty acid elongation, synthesis and degradation of ketone bodies, and fatty acid metabolism.

### Validation of DEGs by qRT-PCR

**Figure 5:**
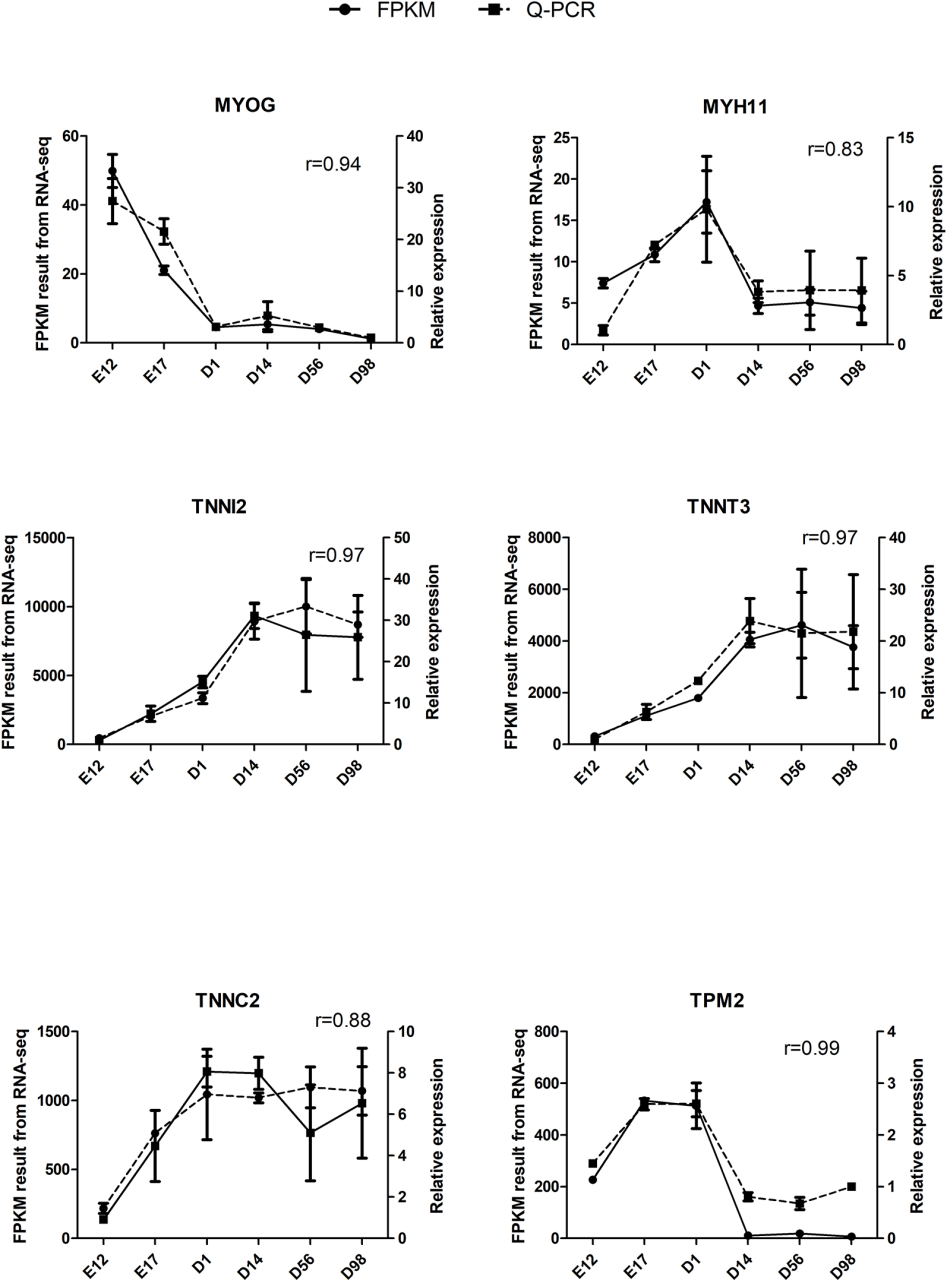
Validation of six DEGs by qRT-PCR. The *r* value represents Pearson’s correlation coefficient between two methods.

The qRT-PCR assays were conducted to validate six selected DEGs from RNA-Seq: *MYOG*, *MYH11*, *TNNI2*, *TNNT3*, *TNNC2*, and *TPM2*. Relative expression changes of qRT-PCR data were highly (*r* = 0.83–0.99) correlated with RNA-Seq data (**Figure 5**), suggesting the reliability of the RNA-Seq approach.

## Discussion

Skeletal muscle growth and development includes a series of closely regulated changes in gene expression level from embryo to adult, and uncovering the gene expression patterns underneath chicken skeletal muscle development contributes to meat production. Previous transcriptome analysis of chicken muscle only concentrated on the embryonic period or adult stage, and few studies have systematically examined the transcriptome of chicken skeletal muscle development from the embryonic period to the growing period. To investigate the mechanisms of skeletal muscle development systematically, we used RNA-Seq to generate extensive cDNA libraries for six developmental stages of chickens from E12 to D98. As shown in **Figure 1** and **Table S2**, a total of 9,447 DEGs were identified in pairwise comparisons between the libraries of breast muscle at the six developmental stages, and the regional differences in gene expression were greatest during the earlier stages of embryo development (E17 versus E12) than the late postnatal stage (D98 versus D56). Previous studies have demonstrated the increase in cell number or muscle fiber number which occurs mainly in embryonic periods as the numbers of muscle fibers were fixed by the day of hatching and then impacted on the postnatal accretion of muscle mass (Smith, 1963; Sporer et al., 2011). Thus, embryonic periods are the critical periods for muscle development and more genes are active in these periods. Six selected DEGs involved in muscle development were validated using qRT-PCR, and the results were consistent with those from RNA-Seq, suggesting reliability of the identified DEGs through RNA-Seq (**Figure 5**).

Since muscle development was accompanied by the differential expression of related genes in different growth periods and our data were also collected at different time points, we used STEM software, which is widely used to study dynamic biological processes (Guo et al., 2016; Ma et al., 2018; Zhan et al., 2018), to investigate the dynamic genetic changes during breast muscle development. We experimented with various numbers of profiles and found that five profiles (combining with three clusters) best captured the expression patterns of DEGs (**Figure 2** and **Table S3**). The genes in a cluster have similar temporal expression patterns and may be involved in the same biological process. Therefore, we performed GO and KEGG analyses to explore the function of the DEGs with similar temporal expression patterns.

For cluster I with downregulated pattern (**Figure 2B** and **Table S3**), 3,926 genes were significantly clustered, which was more than 40% of the total DEGs. These genes were more highly expressed in the early periods of muscle development than the late stages of growth, which further confirmed that the early development might play key roles in muscle growth. GO functional annotation and KEGG analysis both showed that the downregulated genes were significantly enriched in cell proliferation, including DNA replication, cell cycle, cell division, mitotic nuclear division, and DNA repair (**Figures 3A** and **4A**), which was similar to previous research on goat muscle development from gestation to birth which showed that genes with downregulated patterns were also involved in cell proliferation processes (Zhan et al., 2018). Therefore, these results further support the hypothesis that the total number of skeletal myofibers is defined by hyperplasia during embryogenesis. Among these downregulated genes, *CCNA2*, *CCNB2*, and *CDK1*, which encode the cyclins and their cognate cyclin-dependent protein kinases, were not only significantly enriched in the biological process of cell division but also significantly enriched in cell cycle pathway (**Tables S4** and **S7**). Cyclin A2 possesses a unique role in its two-point control of the cell cycle, first by interacting with CDK2 in controlling the G1/S transition into DNA synthesis and then by interacting with CDKs 1 and 2 to control the G2/M entry into mitosis (Li et al., 1998). Previous study has been demonstrated that constitutive expression of cyclin A2 in a transgenic mouse yields robust postnatal cardiomyocyte mitosis and hyperplasia (Chaudhry et al., 2004). Cyclin B2 was also demonstrated to have a regulatory role in chicken breast muscle development (Li et al., 2019). Moreover, CDK1 and CDK2 play integral roles in reducing MyoD activity during myoblast proliferation by phosphorylating MyoD (Kitzmann et al., 1999). These results suggest that the genes with a downregulated pattern of expression play regulatory roles in chicken breast muscle development through the processes involved in the early stages of cell proliferation, and genes related to cyclins and their cognate cyclin-dependent protein kinases may be critical factors in regulating cell proliferation.

For cluster II with an upregulated pattern of expression (**Figure 2C** and **Table S3**), the functional annotation and pathway analysis both showed that these genes were significantly enriched in metabolism such as carbohydrate metabolism, glycolysis/gluconeogenesis, calcium signaling pathway, insulin signaling pathway, and biosynthesis of amino acids (**Figures 3B** and **4B**). A previous study of goat muscle development also found that genes with upregulated patterns of expression were related to metabolic pathways, such as biosynthesis of amino acids, glycolysis/gluconeogenesis, and the TCA cycle (Zhan et al., 2018). Among these genes, *PHKG1*, *PPP1R3C*, and *FBP2* were significantly enriched not only in glycogen biosynthetic process and gluconeogenesis but also in insulin signaling pathway and calcium signaling pathway (**Tables S5** and **S8**). *PHKG1*, as a key factor in insulin signaling and calcium signaling pathways, encodes the catalytic subunit of phosphorylase kinase, which functions in the cascade activation of glycogen breakdown in muscle tissue (Ma et al., 2014). Fructose-1,6-bisphosphatase encoded by *FBP2* catalyzes the hydrolysis of fructose-1,6-bisphosphate to fructose-6-phosphate and inorganic phosphate, which plays a regulatory role in the synthesis of glycogen/glucose. Previous findings point to FBP2 as an important link between calcium-induced muscle contractive and metabolic (glycolytic) activity, mitochondrial function, and cell survival (Pirog et al., 2014). PPP1R3C was also called protein targeting to glycogen (PTG) and regulated glycogen metabolism (Ji et al., 2019). Moreover, glycolytic process and biosynthesis of amino acids pathway were both significantly enriched in *PKLR*, *PGK2*, and *TPI1* (**Tables S5** and **S8**); thus, these genes may be important regulatory switches for protein and energy conversion and ultimately influence muscle development. A number of studies have demonstrated that muscle mass increased by hypertrophy (increased cellular protein content) after hatching and was controlled by synthesis of muscle proteins or their degradation (Braun and Gautel, 2011). Protein and energy metabolism are tightly coupled, and the energy from glycolysis/gluconeogenesis is needed for protein turnover during skeletal muscle development (Duan et al., 2016; Liu et al., 2016). Therefore, these results show that genes involved in metabolism may be critical for postnatal myofiber growth, muscle hypertrophy, and muscle regeneration, and the protein synthesis and energy metabolism of skeletal muscle regulated by insulin signaling pathway and calcium signaling pathway may be important for coordinating muscle development.

Cluster III (profile 25) showed an increase from E17 to D1 and reached a peak at D1, then decreased from D1 to D14 and remained stable from D14 to D98 (**Figure 2D** and **Table S3**). The functional annotation showed that processes and pathways involved in lipid metabolism were significantly enriched, such as fatty acid β-oxidation, lipid homeostasis, fatty acid degradation, and propanoate metabolism (**Figures 3C** and **4C**). Previous studies have demonstrated that lipids stored in the adipocytes during embryonic life are transferred to the muscle fibers and used for growth and energy requirements at the early stage, while muscle again stores lipids in later life (Chartrin et al., 2007; Liu et al., 2016). Interestingly, *ACADL*, *ACAT1*, *HADHA*, *ACADS*, *ECHS1*, and *AUH*, which are significantly enriched in fatty acid beta-oxidation, were active during embryonic life in the present study (**Table S6**), which further demonstrated that the lipids were the important energy source for muscle development and growth at the early stage. Moreover, some of these genes were also the key regulatory molecules for intramuscular fat (IMF) deposition. For example, *ACADL* and *HADHA* have been identified as candidate biomarkers for IMF deposition in Cobb and Beijing-You chickens (Liu et al., 2017a), and it was interesting that their expression patterns in Shouguang chickens were similar to Cobb and Beijing-You chickens. Moreover, *ACAT1* expression was significantly lower in muscle of AA chickens with low IMF content than in Beijing-You chickens with abundant IMF (Liu et al., 2018), suggesting that *ACAT1* may contribute to IMF deposition. These results suggest that genes involved in lipid metabolism, and especially those related to fatty acid beta-oxidation, play important roles in early muscle development and deposition of IMF.

## Conclusion

In the present study, we systematically identified DEGs and investigated their temporal expression profiles during chicken breast muscle development from E12 to D98. A total of 9,447 DEGs were identified in chicken breast muscle and showed three significantly different expression patterns. Functional enrichment analysis suggests that genes with downregulated patterns contribute to cell proliferation processes, while genes with upregulated patterns are mainly involved in metabolism. Genes related to lipid metabolism change dramatically around the time of birth, which may play important roles in early muscle development and deposition of IMF. In summary, our study will facilitate understanding of the mechanisms underlying the phenotype that myofiber hyperplasia occurs predominantly during embryogenesis and hypertrophy occurs mainly after birth at the transcriptional level. These findings elucidate the regulatory mechanisms involved in chicken breast muscle development.

## Data Availability Statement

The raw sequence data reported in this paper have been deposited in the Genome Sequence Archive in BIG Data Center, Beijing Institute of Genomics (BIG), Chinese Academy of Sciences and is publicly accessible at http://bigd.big.ac.cn/gsa (accession no CRA001773).

## Ethics Statement

All experiments were approved by the Animal Care Committee of the Shandong Academy of Agricultural Sciences (Ji’nan, China). The experimental procedures with chickens were performed according to the Guidelines for Experimental Animals established by the Ministry of Science and Technology (Beijing, China).

## Author Contributions

JL and QL performed experiments and data analysis and draft writing. FL, YZ, JG, and WL contributed to animal experiments and data analysis. HH and DC designed experiments and supervised and coordinated the study. All authors reviewed the manuscript.

## Funding

The research was supported by grants from Natural Science Foundation of Shandong province (ZR2019BC077), the Earmarked Fund for Modern Agro-industry Technology Research System (CARS-41), Jinan Layer Experiment Station of China Agriculture Research System (CARA-40-S12), Agricultural Scientific and Technological Innovation Project of Shandong Academy of Agricultural Sciences (CXGC2016A04), Shandong Provincial Key Laboratory of Special Construction Project (SDKL201810), and Construction of Subjects and Teams of Institute of Poultry Science (CXGC2018E11).

## Conflict of Interest

The authors declare that the research was conducted in the absence of any commercial or financial relationships that could be construed as a potential conflict of interest.

## Abbreviations

ACADL, acyl-CoA dehydrogenase, long chain; ACADS, acyl-CoA dehydrogenase, C-2 to C-3 short chain; ACAT1, acetyl-CoA acetyltransferase 1; AUH, AU RNA binding methylglutaconyl-CoA hydratase; CCNA2, cyclin A2; CCNB2, cyclin B2; CDK1, cyclin-dependent kinase 1; ECHS1, enoyl-CoA hydratase, short chain 1; FBP2, fructose-bisphosphatase 2; HADHA, hydroxyacyl-CoA dehydrogenase/3-ketoacyl-CoA thiolase/enoyl-CoA hydratase (trifunctional protein), alpha subunit; PGK2, phosphoglycerate kinase 2; PHKG1, phosphorylase kinase catalytic subunit gamma 1; PKLR, pyruvate kinase, liver and RBC; PPP1R3C, protein phosphatase 1 regulatory subunit 3C; TPI1, triosephosphate isomerase 1.

## References

[B1] Braun T. Gautel M. (2011). Transcriptional mechanisms regulating skeletal muscle differentiation, growth and homeostasis. Nat. Rev. Mol. Cell Biol. 12 (6), 349. 10.1038/nrm3118 21602905

[B2] Chartrin P. Bernadet M. Guy G. Mourot J. Hocquette J.-F. Rideau N. (2007). Do age and feeding levels have comparable effects on fat deposition in breast muscle of mule ducks? Animal 1 (1), 113–123. 10.1017/S1751731107658029 22444214

[B3] Chaudhry H. W. Dashoush N. H. Tang H. Zhang L. Wang X. Wu E. X. (2004). Cyclin A2 mediates cardiomyocyte mitosis in the postmitotic myocardium. J. Biol. Chem. 279 (34), 35858–35866. 10.1074/jbc.M404975200 15159393

[B4] Davis R. V. Lamont S. J. Rothschild M. F. Persia M. E. Ashwell C. M. Schmidt C. J. (2015). Transcriptome analysis of post-hatch breast muscle in legacy and modern broiler chickens reveals enrichment of several regulators of myogenic growth. PloS One 10 (3), e0122525. 10.1371/journal.pone.0122525 25821972 PMC4379050

[B5] DennisG.ShermanB. T.HosackD. A.YangJ.GaoW.LaneH. C. (2003). DAVID: database for annotation, visualization, and integrated discovery. Genome Biol. 4 (9), R60. 10.1186/gb-2003-4-9-r60 12734009

[B6] Duan Y. Li F. Li Y. Tang Y. Kong X. Feng Z. (2016). The role of leucine and its metabolites in protein and energy metabolism. Amino Acids 48 (1), 41–51. 10.1007/s00726-015-2067-1 26255285

[B7] Ernst J. Bar-Joseph Z. (2006). STEM: a tool for the analysis of short time series gene expression data. BMC Bioinf. 7 (1), 191. 10.1186/1471-2105-7-191 PMC1456994 16597342

[B8] Fridolfsson A.-K. Ellegren H. (1999). A simple and universal method for molecular sexing of non-ratite birds. J. Avian Biol. 30 (1), 116–121. 10.2307/3677252

[B9] Gao Y. Tu Y. Tong H. Wang K. Tang X. Chen K. (2008). Genetic variation of indigenous chicken breeds in China and a Recessive White breed using AFLP fingerprinting. S. Afr. J. Anim. Sci. 38 (3), 193–200. 10.4314/sajas.v38i3.4129

[B10] Guo J. Zhao W. Zhan S. Li L. Zhong T. Wang L. (2016). Identification and expression profiling of miRNAome in goat longissimus dorsi muscle from prenatal stages to a neonatal stage. PloS One 11 (10), e0165764. 10.1371/journal.pone.0165764 27798673 PMC5087842

[B11] Ji X. Wang S. Tang H. Zhang Y. Zhou F. Zhang L. (2019). PPP1R3C mediates metformin-inhibited hepatic gluconeogenesis. Metabolism 10.1016/j.metabol.2019.06.002 31181215

[B12] Kim D. Langmead B. Salzberg S. L. (2015). HISAT: a fast spliced aligner with low memory requirements. Nat. Methods 12 (4), 357. 10.1038/nmeth.3317 25751142 PMC4655817

[B13] Kitzmann M. Vandromme M. Schaeffer V. Carnac G. Labbé J.-C. Lamb N. (1999). cdk1-and cdk2-mediated phosphorylation of MyoD Ser200 in growing C2 myoblasts: role in modulating MyoD half-life and myogenic activity. Mol. Cell. Biol. 19 (4), 3167–3176. 10.1128/MCB.19.4.3167 10082583 PMC84110

[B14] Li J.-M. Poolman R. A. Brooks G. (1998). Role of G1 phase cyclins and cyclin-dependent kinases during cardiomyocyte hypertrophic growth in rats. Am. J. Physiol.-Heart Circ. Physiol. 275 (3), H814–H822. 10.1152/ajpheart.1998.275.3.H814 9724284

[B16] Li Z. Ouyang H. Zheng M. Cai B. Han P. Abdalla B. A. (2017). Integrated analysis of long non-coding RNAs (LncRNAs) and mRNA expression profiles reveals the potential role of LncRNAs in skeletal muscle development of the chicken. Front. Physiol. 7, 687. 10.3389/fphys.2016.00687 28119630 PMC5220077

[B15] Li Y. Chen Y. Jin W. Fu S. Li D. Zhang Y. (2019). Analyses of MicroRNA and mRNA Expression Profiles Reveal the Crucial Interaction Networks and Pathways for Regulation of Chicken Breast Muscle Development. Front. Genet. 10, 197. 10.3389/fgene.2019.00197 30936892 PMC6431651

[B17] Liu J. Fu R. Liu R. Zhao G. Zheng M. Cui H. (2016). Protein profiles for muscle development and intramuscular fat accumulation at different post-hatching ages in chickens. PloS One 11 (8), e0159722. 10.1371/journal.pone.0159722 27508388 PMC4980056

[B18] Liu L. Cui H. Fu R. Zheng M. Liu R. Zhao G. (2017a). The regulation of IMF deposition in pectoralis major of fast-and slow-growing chickens at hatching. J. Anim. Sci. Biotechnol. 8 (1), 77. 10.1186/s40104-017-0207-z 29026539 PMC5623058

[B20] Liu R. Wang H. Liu J. Wang J. Zheng M. Tan X. (2017b). Uncovering the embryonic development-related proteome and metabolome signatures in breast muscle and intramuscular fat of fast-and slow-growing chickens. BMC Genomics 18 (1), 816. 10.1186/s12864-017-4150-3 29061108 PMC5653991

[B19] Liu L. Cui H. Zheng M. Zhao G. Wen J. (2018). Comparative analysis of differentially expressed genes related to triglyceride metabolism between intramuscular fat and abdominal fat in broilers. Br. Poult. Sci. 59 (5), 514–520. 10.1080/00071668.2018.1483573 29939074

[B21] Livak K. J. Schmittgen T. D. (2001). Analysis of relative gene expression data using real-time quantitative PCR and the 2– ΔΔCT method. Methods 25 (4), 402–408. 10.1006/meth.2001.1262 11846609

[B22] Ma J. Yang J. Zhou L. Ren J. Liu X. Zhang H. (2014). A splice mutation in the PHKG1 gene causes high glycogen content and low meat quality in pig skeletal muscle. PloS Genet. 10 (10), e1004710. 10.1371/journal.pgen.1004710 25340394 PMC4207639

[B23] Ma Y. Feng S. Wang X. Qazi I. H. Long K. Luo Y. (2018). Exploration of exosomal microRNA expression profiles in pigeon ‘Milk’during the lactation period. BMC Genomics 19 (1), 828. 10.1186/s12864-018-5201-0 30458711 PMC6245878

[B24] Ouyang H. Wang Z. Chen X. Yu J. Li Z. Nie Q. (2017). Proteomic analysis of chicken skeletal muscle during embryonic development. Front. Physiol. 8, 281. 10.3389/fphys.2017.00281 28533755 PMC5420592

[B25] Pertea M. Pertea G. M. Antonescu C. M. Chang T.-C. Mendell J. T. Salzberg S. L. (2015). StringTie enables improved reconstruction of a transcriptome from RNA-seq reads. Nat. Biotechnol. 33 (3), 290. 10.1038/nbt.3122 25690850 PMC4643835

[B26] Pirog M. Gizak A. Rakus D. (2014). Changes in quaternary structure of muscle fructose-1, 6-bisphosphatase regulate affinity of the enzyme to mitochondria. Int. J. Biochem. Cell Biol. 48, 55–59. 10.1016/j.biocel.2013.12.015 24412565

[B27] Robinson M. D. McCarthy D. J. Smyth G. K. (2010). edgeR: a Bioconductor package for differential expression analysis of digital gene expression data. Bioinformatics 26 (1), 139–140. 10.1093/bioinformatics/btp616 19910308 PMC2796818

[B28] Smith J. H. (1963). Relation of body size to muscle cell size and number in the chicken. Poult. Sci. 42 (2), 283–290. 10.3382/ps.0420283

[B29] Sporer K. R. Tempelman R. J. Ernst C. W. Reed K. M. Velleman S. G. Strasburg G. M. (2011). Transcriptional profiling identifies differentially expressed genes in developing turkey skeletal muscle. BMC Genomics 12 (1), 143. 10.1186/1471-2164-12-143 21385442 PMC3060885

[B30] Xie C. Mao X. Huang J. Ding Y. Wu J. Dong S. (2011). KOBAS 2.0: a web server for annotation and identification of enriched pathways and diseases. Nucleic Acids Res. 39 (suppl_2), W316–W322. 10.1093/nar/gkr483 21715386 PMC3125809

[B31] Ylihärsilä H. Kajantie E. Osmond C. Forsen T. Barker D. Eriksson J. (2007). Birth size, adult body composition and muscle strength in later life. Int. J. Obesity 31 (9), 1392. 10.1038/sj.ijo.0803612 17356523

[B32] Zhan S. Zhao W. Song T. Dong Y. Guo J. Cao J. (2018). Dynamic transcriptomic analysis in hircine longissimus dorsi muscle from fetal to neonatal development stages. Funct. Integr. Genomics 18 (1), 43–54. 10.1007/s10142-017-0573-9 28993898

